# QSAR Models for Sweetness: Can They Shape the Future of Nutritional Safety?

**DOI:** 10.3390/foods15091481

**Published:** 2026-04-23

**Authors:** Alla P. Toropova, Andrey A. Toropov, Ivan Raŝka, Maria Raŝkova, Patnala Ganga Raju Achary

**Affiliations:** 1 Department of Environmental Health Sciences, Istituto di Ricerche Farmacologiche Mario Negri IRCCS, Via Mario Negri 2, 20156 Milan, Italy; andrey.toropov@marionegri.it; 2 3rd Medical Department, 1st Faculty of Medicine, Charles University in Prague and General University Hospital, U Nemocnice 1, 12808 Prague, Czech Republic; ivan.raska@vfn.cz (I.R.J.); maria.raskova@vfn.cz (M.R.); 3 Department of Chemistry, Faculty of Engineering & Technology (ITER), Siksha ‘O’ Anusandhan Deemed to Be University, Bhubaneswar 751030, Odisha, India; pgrachary@soa.ac.in

**Keywords:** food safety, nutrition, public health, sweetness, QSAR, Monte Carlo method, SMILES, Las Vegas algorithm

## Abstract

Food safety, nutrition, and public health are actual economic and medical problems. Sweetness is an important feature of food technology. Models for the sweetness of special organic compounds used in the food industry are suggested. The models are built using the CORAL software. New statistical coefficients of predictive potential are studied. These are the index of ideality of correlation (IIC) and correlation intensity index (CII). The effectiveness of using the IIC and CII has been tested in simulated sweetness via Monte Carlo optimization of correlation weights for molecular features extracted from Simplified Molecular Input Line Entry System (SMILES) strings. Both factors have been shown to improve the model’s statistical quality on the calibration and validation sets. However, this is accompanied by a decrease in the statistical quality of the training sets.

## 1. Introduction

The escalating global prevalence of obesity, type II diabetes, and cardiovascular diseases represents a deep public health and economic challenge [1]. A significant contributing factor to this crisis is the excessive consumption of added sugars, particularly sucrose and high-fructose corn syrup, prevalent in processed foods and sugar-sweetened beverages [2]. In response, the food and beverage industry has increasingly turned to non-nutritive (non-caloric) and low-calorie sweeteners to provide the desirable sensory attribute of sweetness without the associated caloric burden [3]. These sweeteners, derived from both natural sources and synthetic processes, are, however, subjects of ongoing scientific and public debate regarding their long-term health effects, safety profiles, and metabolic impacts [4]. Consequently, the development of novel, safer, and more efficient sweetening agents is a pressing priority. The process of discovering and approving new food additives is traditionally expensive and time-consuming, relying heavily on animal testing and human sensory panels.

Key aspects of sweetener regulation are quite complex since including (i) list of authorized sweeteners; (ii) European Food Safety Authority (EFSA) is carrying out periodic safety re-evaluation is currently re-evaluating the safety of all food additives approved before 2009, including sweeteners, to ensure their continued safety; (iii) labelling requirements; (iv) restrictions; and (v) warnings [5,6,7,8].

Several years ago, a compromise between Codex Alimentarius members, the Food and Agriculture Organization (FAO), and the World Health Organization (WHO) resulted in the development of common standards for food additives, allowing the use of sweeteners to reduce energy value or improve taste in certain food categories [7,9,10].

There exists a critical need for reliable computational tools to screen and prioritize candidate molecules before they enter the costly experimental pipeline.

Quantitative Structure–Activity Relationship (QSAR) modeling presents a powerful in silico methodology to take such challenges. The fundamental principle of QSAR is that a compound’s biological activity (here, ‘sweetness’) can be quantitatively related to its molecular structure, known as descriptors [11]. By building a statistical model based on a training set of compounds with known activities, it becomes possible to predict the activity of new, untested compounds. Sweetness intensity is most quantified as relative sweetness, defined as the concentration of a sucrose solution perceived to be equally sweet to a solution of the compound under study. Given the immense range of sweetness potency—varying over several orders of magnitude from sucrose to potent compounds like saccharin or aspartame—it is conventional and practical to express this property in logarithmic units, log(S), where S is the relative sweetness compared to sucrose [12,13]. This log(S) endpoint is highly agreeable to QSAR analysis [14,15].

Recent developments in cheminformatics have extended the toolkit available for QSAR. Beyond traditional molecular descriptors, simplified molecular line entry systems (SMILES) have emerged as an effective means of representing molecular structure for algorithmic processing [16]. Software packages like CORAL utilize SMILES notation, applying Monte Carlo optimization techniques to generate correlation weights (CW) for specific molecular attributes, thereby building transparent and interpretable QSAR models [17,18]. The core of any QSAR model-building process is an optimization algorithm that selects the most relevant molecular descriptors and assesses their contributions to the model. This algorithm is guided by an objective or target function that it seeks to maximize or minimize. The choice of this target function is paramount, as it fundamentally dictates the model’s characteristics, including its predictive accuracy, robustness, and generalizability to all data. Traditional optimization often relied on maximizing the correlation coefficient (R^2^) for the training set. However, this approach can lead to overfitting, in which a model performs exceptionally well on its training data but fails to predict the performance of external compounds accurately. To overcome this limitation, more sophisticated target functions have been developed.

Two prominent examples are:

- The Index of Ideality of Correlation (IIC): this index is designed to balance the correlation coefficient with the model’s error, penalizing models where the error of prediction is not randomly distributed. It aims to produce a more “ideal” and reliable correlation [19].
- The Correlation Intensity Index (CII): this index is proposed to intensify the correlation and, crucially, to enhance the model’s predictive performance on external validation sets, thereby improving its practical utility [20].

The comparative efficacy of these two advanced target functions for building predictive models of complex sensory properties, such as sweetness, remains an open and highly relevant question.

Questions about model validation have been raised and discussed many times [21,22,23,24,25,26]. However, good statistics for the training set are often accompanied by poor statistics on the validation set [27], even if a wide variety of molecules are included in the training set [28].

Both the IIC and the CII range from 0 to 1. However, the IIC is an attempt to develop a means of avoiding the situation in which a high correlation coefficient (determination coefficient) is accompanied by a high standard deviation (or mean absolute error). Such situations arise from mismatches in the slopes of the regression lines between the training and validation samples. Thus, the IIC forces the regression line to be as close as possible to the diagonal on the experimental-versus-calculated values diagram. The direction of the CII is somewhat different. In terms of “supporters” and “opponents” of correlation in the same coordinates (experimental versus calculated values for the endpoint under consideration), optimization that maximizes the objective function is intended to reduce overall rejection. From a geometric perspective, such optimization reduces the number of points (experiments, calculations) that contribute to the overall correlation.

The present research aims to investigate a systematic comparative analysis of QSAR models for the log(S) endpoint. The primary objective is to evaluate the performance, stability, and predictive power of models generated using two different target functions: one based on the IIC (TF_IIC_) and another based on the CII (TF_CII_).

We hypothesize that while both advanced functions will yield models superior to those built with traditional criteria, the CII-based target function will produce models with enhanced external predictive ability due to its explicit design objective of intensifying correlation for validation compounds. The findings of this study will provide valuable guidance for cheminformaticians and food scientists in selecting optimal strategies for the in silico design and screening of novel sweet compounds.

## 2. Materials and Methods

### 2.1. Data Curation

Data on 315 log(S) taken from [29]. However, 56 duplicates were detected for the dataset. The dataset of 289 compounds remains after removing 26 compounds (one from each duplicate pair). These compounds were distributed into active training set (A), passive training set (P), calibration set (C), and validation set (V). This distribution was carried out using the Las Vegas algorithm. The idea of the Las Vegas algorithm can be expressed by the words “if I play again and again, I’m sure to win someday.” The distribution was done under two aims: (i) the number of compounds in each set is approximately the same; and (ii) the statistical quality of the calibration set is preferable (a good statistical quality model for the calibration set). In practice, it was the best model for the calibration set across 10 Monte Carlo runs with different splits using CORAL software-2025 (www.insilico.eu/coral, accessed on 20 April 2026).

The actions described require that the training and validation splits under consideration be significantly diverse. Table 1 and Table 2 confirm sufficient variability between the splits under consideration. Given that the Las Vegas algorithm was used to select the splits considered here, these splits are not entirely random, although they are completely random before the Las Vegas algorithm is run.

### 2.2. Model

The model for sweetness is the following


(1) $$\log\left(S\right)=C_{0}+C_{1}\times DCW(T,N)$$

DCW(T,N) is an optimal descriptor calculated with correlation weights (CW) of active (not rare) SMILES attributes and correlation weights of Nearest Neighbor Codes (NNC) [30]

(2) $$DCW\left(T,N\right)=\sum {CW(S}_{k})+\sum CW\left({SS}_{k}\right)+\sum CW({NNC}_{k})$$

CW(S_k_) is the correlation weight of a SMILES atom. SMILES-atom is one symbol, or several symbols which cannot be examined separately; CW(SS_k_) is the correlation weight of a pair of connected SMILES-atoms. For instance, in the case of SMILES that represented formaldehyde C = O, S_k_ = {C’,‘=’,‘O’}; SS_k_ = {‘C=’,‘=O’}.

NNC_k_ is the nearest neighbor code for the k-th vertex [30]. NNC_k_ is calculating with a hydrogen-suppressed graph as follows

(3) $${NNC}_{k}=100\times N_{T}+10\times N_{C}+N_{x}$$

N_T_ is the total number of neighbors for the k-th vertex; N_C_ is the number of neighbors that are carbon atoms; and N_X_ is the number of vertices representing non-carbon atoms.

T and N are parameters of Monte Carlo optimization. T is an integer to define rare and active (non-rare) SMILES attributes. If T = 3, then SMILES attributes with a frequency in the active training set less than 3 are rare and are not involved in the simulation process. N is the number of epochs in the Monte Carlo optimization; here, N = 15.

### 2.3. Monte Carlo Optimization

The optimization starts by splitting the available data into four subsets: active training set (A), passive training set (P), calibration set (C), and validation set (V). This is carried out using the Las Vegas algorithm (by selecting a split that is favorable to the calibration set). Next, the correlation weights for the active SMILES attributes are selected to maximize the objective function. Two different target functions are compared here, TF_1_ and TF_2_, which are calculated as

(4) $${TF}_{IIC}=R_{A}^{2}+R_{P}^{2}-\left|R_{A}^{2}-R_{P}^{2}\right|+IIC\times 0.3$$

(5) $${TF}_{CII}=R_{A}^{2}+R_{P}^{2}-\left|R_{A}^{2}-R_{P}^{2}\right|+CII\times 0.3$$

R^2^_A_ and R^2^_P_ are the coefficients of determination on the active and passive training sets, respectively. The coefficient 0.3 was selected empirically.

The IIC is the index of ideality of correlation [19,31]. The IIC is calculated with data on the calibration set as follows:

(6) IICC=rCmin(MAEC−,MAEC+)max(MAEC−,MAEC+)

(7) $$\min\left(x,y\right)=\left\{\begin{array}{c} x,\;if\;x<y\\ y,\mathrm{otherwise} \end{array}\right.$$

(8) $$\max\left(x,y\right)=\left\{\begin{array}{c} x,\;if\;x>y\\ y,\;\mathrm{otherwise} \end{array}\right.$$

(9) MAEC−=1N−∑|∆k|,N is the number of ∆k−<0

(10) MAEC+=1N+∑|∆k|,N is the number of ∆k+≥0

(11) $$\Delta_{k}={observed}_{k}-{calculated}_{k}$$

The observed and calculated values correspond to the endpoint.

The CII is the correlation intensity index [20]. The index is calculated as

(12) $${CII}_{C}=1-\sum {Protest}_{k}$$

(13) $${Protest}_{k}=\left\{\begin{array}{cc} R_{k}^{2}-R^{2}, & if\;R_{k}^{2}-R^{2}>0\\ 0, & otherwise \end{array}\right.$$

The R^2^ is the coefficient of determination for a set containing n substances. The R^2^_k_ is the correlation coefficient for n − 1 substances of a set, after removing the k-th substance. Hence, if the (R^2^_k_ − R^2^) is larger than zero, the k-th substance is an “oppositionist” for the correlation between experimental and predicted values of the set. A small sum of “protests” means a more “intensive” correlation.

Checking the percentage of identity among different data splits is a crucial step in evaluating the robustness and generalizability of predictive models, particularly in cheminformatics and QSAR (Quantitative Structure–Activity Relationship) studies. This analysis helps assess the degree of overlap between compound sets used at different stages of model development, specifically the active training sets and the calibration sets across different splits. High similarity between splits may indicate data leakage or overfitting, while low overlap suggests that models are being validated on truly independent data, thereby increasing confidence in their external predictive performance.

For the above reasons, two types of models were examined: IIC models and CII models, each optimized using distinct target functions—TF_IIC_ and TF_CII_, respectively. To quantify the inter-split compound similarity, identity percentages were calculated and presented in two symmetrical matrices: Table 1 lists the IIC models, whereas Table 2 lists the CII models. These tables present pairwise comparisons of seven data splits (labeled 1 through 7), with each cell showing the percentage of compounds shared between the corresponding splits. In both tables, the upper triangle entries reflect the percentage of identical compounds present in the active training sets of the respective split pairs. Conversely, the lower triangle entries represent the percentage of overlapping compounds in the calibration sets—the subset of data used to tune and validate model performance during development. The diagonal elements are uniformly 100%, as they represent self-comparisons of each split.

In the IIC models (Table 1), the active training set comparisons (upper triangle) show moderate to high overlap. For instance, split 4 and split 5 share 72.2% of compounds in their calibration sets (lower triangle, row 5, column 4), indicating significant data overlap, which could raise concerns about over-optimistic calibration results. However, other pairs, such as splits 4 and 6, show only 19.4% overlap in calibration sets, suggesting more independent validation conditions. The variation across the matrix highlights the importance of probing all pairwise relationships rather than relying on overall statistics alone.

The CII models (Table 2) display a notably different pattern. Several comparisons, particularly those involving splits 3, 5, and 6, reveal high percentages of shared compounds across both the training and calibration sets. For example, splits 5 and 6 share 73.6% of compounds in their calibration sets (lower triangle, row 6, column 5), and their active training sets share 73.6% as well (upper triangle, row 5, column 6). Such high overlap suggests a strong potential for overfitting and limited independence across these splits, which may inflate perceived model performance. In contrast, splits 1 and 4 show only 18.1% overlap in calibration sets, indicating more conservative data partitioning.

### 2.4. Construction of Models

To construct robust and statistically reliable models, a rigorous optimization protocol was implemented. Specifically, 10 independent Monte Carlo-based optimization runs were conducted for each modeling strategy. The Monte Carlo approach randomly samples the feature space and data partitions to explore diverse model configurations, thereby reducing selection bias and enhancing the likelihood of identifying globally optimal solutions. From the 10 models generated in each series, the single best-performing model, based on predictive accuracy on the calibration set, was selected for further analysis and reporting. This selection criterion ensures that the chosen model demonstrates optimal internal consistency and calibration performance, although care must be taken to confirm that this does not come at the expense of overfitting—particularly given the observed data overlaps in some splits. The first series was carried out with the target function TF_IIC_. The second series was carried out with the target function TF_CII_.

### 2.5. Applicability Domain

The applicability domain of the considered models is defined as a statistical defect [29] and assessed by examining the representativeness of SMILES attributes for a given SMILES (or the corresponding molecular system). Defects for SMILES features (which represent molecular features) are calculated as:

(14) $$d_{k}=\frac{\left|P(A_{k})-{P^{\prime }(A}_{k})\right|}{N\left(A_{k}\right)+N^{\prime }(A_{k})}+\frac{\left|P(A_{k})-{P^{\prime \prime }(A}_{k})\right|}{N\left(A_{k}\right)+N^{\prime \prime }(A_{k})}+\frac{\left|{P^{\prime }(A}_{k})-{P^{\prime \prime }(A}_{k})\right|}{N^{\prime }\left(A_{k}\right)+N^{\prime \prime }(A_{k})}$$

where P(A_k_), P′(A_k_) P″(A_k_) are the probabilities of A_k_ in the active training, passive training, and calibration sets, respectively; N(A_k_), N′(A_k_), and N″(A_k_) are the frequencies of A_k_ in the active training set, passive training set, and calibration set. The statistical SMILES-defects (D_j_) are calculated as:

(15) $$D_{j}=\sum_{k=1}^{NA}d_{k}$$

where NA is the number of non-blocked SMILES attributes in the SMILES.

A SMILES falls into the applicability domain if


(16) $$Dj<2\times \bar{D}$$

$\bar{D}$ is average on the list of SMILES attribute defects {Dj}.

Table 3 shows that removing outliers detected using inequality 16 improves the statistical quality of the corresponding models. Although the coefficient of determination deteriorates slightly, the mean absolute error for the case of the applicability domain is improved for all seven splits considered.

### 2.6. Mechanistic Interpretation

The approach used here can be a source of mechanistic interpretation if one carries out several starts of the described Monte Carlo optimization. In these computational experiments, one can select some active attributes with permanently positive correlation weights, along with attributes with permanently negative weights. The first can be considered promoters of increased sweetness, the second of decreased sweetness.

## 3. Results

### 3.1. The Monte Carlo Optimization Using TF_IIC_

Table 3 provides a comprehensive overview of the statistical characteristics derived from computational experiments with the TF_IIC_ target functions. These models were developed and evaluated across seven different data splits (labeled 1 through 7), with each split consisting of four subsets: the active training set (A), passive training set (P), calibration set (C), and validation set (V). The results are reported for a consistent number of compounds (n = 72 or 73, depending on the subset) to ensure comparability across splits and modeling conditions. Supplementary Materials contain corresponding technical details on the TF_IIC_ model for split 1 in Table S1.

### 3.2. The Monte Carlo Optimization Using TF_CII_

Table 4 presents a detailed analysis of the data, revealing that the models employ TF_CII_. The target function consistently outperforms those based on TF_IIC_. These models were developed and evaluated across seven different data splits (labeled 1 through 7), with each split consisting of four subsets: the active training set (A), passive training set (P), calibration set (C), and validation set (V). The results are reported for a consistent number of compounds (n = 72 or 73, depending on the subset) to ensure comparability across splits and modeling conditions. Supplementary Materials include corresponding technical details on the TF_CII_ model for split 1 in Table S2.

### 3.3. Comparison of Models Obtained Using TF_IIC_ and TF_CII_

The predictive potential of TF_CII_ models is greater than that of TF_IIC_ models. This is most clear in the average values of key statistical parameters, especially the validation set R^2^ (R^2^_V_), which reflects the proportion of variance in the response variable explained by the model on unseen data.

For TF_CII_ models, the average R^2^ across all validation sets is 0.915 ± 0.005, indicating an exceptionally high degree of predictive power and stability. In contrast, models based on TF_IIC_ achieve a notably lower average R^2^ of 0.862 ± 0.028, with greater variability across splits—this broader standard deviation suggests less consistency in performance under different data partitioning schemes.

Further inspection of Table 4 and Table 5 highlights other important performance metrics that reinforce this conclusion. For instance, the Q^2^ values—representing internal cross-validation performance—are generally higher for TF_CII_ models, particularly in calibration sets (C). In several splits (e.g., split 4 and split 5), TF_CII_ achieves Q^2^ values exceeding 0.86, with split 4 reaching an impressive 0.8670. Moreover, advanced validation criteria such as Q^2^F_1_, Q^2^F_2_, Q^2^F_3_, and <Rm2>, which assess the reliability and external predictive performance of quantitative structure–activity relationship (QSAR) models, also favor TF_CII_. For example, in split 4, TF_CII_ produces Q^2^F_1_ = 0.8791, Q^2^F_2_ = 0.8667, Q^2^F_3_ = 0.9377, and <Rm2> = 0.8254, all of which surpass corresponding values for TF_IIC_ models in the same split (Table 4). 

## 4. Discussion

### 4.1. Statistical Quality and Reproducing

A fundamental indicator of a model’s statistical quality is the coefficient of determination. However, if we consider a QSAR model as a random event observed for random splits into a training set (here, the “traditional” training set is structured into active and passive training sets and a calibration set) and a validation set, then, in addition to the coefficient of determination, its dispersion, observed for a group of random splits, is important.

The most significant difference is the coefficient of determination for the validation set. For the objective function of the TF_IIC_, the average coefficient of determination across the validation sets is 0.862, with a standard deviation of 0.028. For the TF_CII_ objective function, the average coefficient of determination across the validation sets is 0.915, with a standard deviation of 0.005. Thus, it is worth noting that the use of the TF_CII_ not only improves the statistical quality of the model for the validation set but, equally importantly, the dispersion of the determination coefficient for the validation sets is significantly lower than that observed with the TF_IIC_. Thus, the predictive potential of the approach based on Monte Carlo optimization with the target function TF_CII_ is not only more accurate but also more stable.

### 4.2. Mechanistic Interpretation

All models are wrong [34], but they sometimes are useful. In practice, they are simple models. Consequently, it is important to select most simple models. Models considered here were calculated using SMILES and a hydrogen-suppressed graph. It is not necessary to use any other descriptors or physicochemical parameters. The average number of outliers, according to the statistical defect, is about 12 (averaged across seven different splits).

Table 6 presents the mechanistic interpretation of promoters of sweetness increase and decrease. It is necessary to consider the frequencies of molecular features across the active, passive training, and calibration sets. The lists of promoters of sweetness increase and decrease contain a considerable portion of NNC, which confirms the value of informativity for the development of the described models. Promoters of increase for sweetness are carbon atoms which have three neighbors, including two carbon atoms and some additional atoms, which is represented by NNC-C...321 (it is not hydrogen because hydrogen suppressed graph considered here); a carbon atom connected with two carbon atoms is represented by NNC-C...220; an oxygen atom connected with two carbon atoms is represented by NNC-O...220. Other promoters of increase for sweetness are double bonds represented by ‘=’, and nitrogen atoms (‘N’). Promoters of decrease for sweetness are the presence of oxygen (in general); atom oxygen connected with one carbon atom (NNC-O...011); as well as a carbon atom connected with three carbon atoms.

The presence of cycles (C...1), according to Table 6, also moderately increases sweetness. Regarding the 3D geometry of molecules, these features are underrepresented in the sample of compounds examined, making it likely that their use as drivers of increased sweetness is statistically unreliable.

It should be noted that, due to the low representation of compounds with pronounced 3D molecular features, a significant portion of such compounds are classified as outliers according to their statistical defects [29].

**Figure 1:**
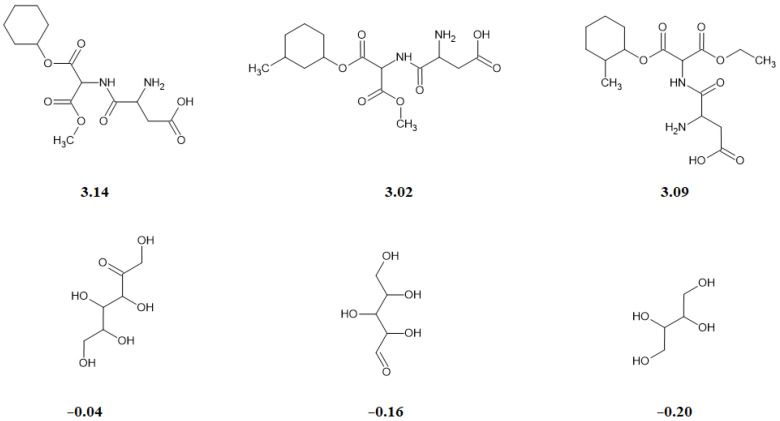
A collection of compounds with different levels of sweetness.

Thus, according to the models discussed, when searching for compounds capable of acting as sweeteners, preference should be given to compounds with a moderate number of rings, a predominance of carbon and nitrogen atoms, and a small number of oxygen atoms. The absence of nitrogen should be interpreted as a sign of reduced sweetness (Figure 1).

### 4.3. Comparison with Models from the Literature

Genetic Function Approximation and artificial neuron networks were examined in [14]. Both approaches give approximately the same predictive potential [14]. Augmented multivariate image analysis was applied to establish a quantitative structure–property relationship (Aug-MIA-QSPR) for sweetness [15].

Comparison with the model of sweetness suggested in [29] (average determination coefficient for validation set in [29] is 0.87, whereas 0.915 here) shows that the Las Vegas algorithm gives the possibility to improve the statistical quality of prediction at least for seven splits of available data into the active and passive training sets, calibration sets, and validation sets. It is worth noting that splitting the data into training and validation sets provides an important argument for trusting the approach.

In addition, the computer experiments described show that, in the development of the sweetness model, the CII is more informative than the IIC. Table 7 contains the comparison of published sweetness models. One can see that the models suggested here are quite comparable to those in the literature.

The approach proposed here allows us to explore several new aspects of QSAR analysis. Such computer experiments can provide a basis for distinguishing between molecular features useful for model construction and those that can undermine the model’s statistical quality, thereby reducing its predictive potential. It should be noted that IIC and CII have found some application in QSAR analysis of various endpoints [35,36,37,38,39,40,41,42,43,44,45]. Thus, the proposed approach can serve as a tool for treating nutrition and sweetness as algorithmic tasks that govern the construction of public health databases.

## 5. Conclusions

The Las Vegas algorithm facilitates a more expansive and robust framework for evaluating QSPR/QSAR models. It enables researchers to efficiently discriminate between molecular features that are truly useful for constructing a predictive model and those that are merely statistical noise or, worse, undermine the model’s generalizability. The results definitively show that for modeling sweetness, the minimalist approach based on SMILES and graph connectivity is not only sufficient but also highly effective, and that the CII is a more suitable choice than IIC for optimizing model quality. This work opens new avenues for QSAR by providing a powerful method for feature selection and model validation. However, it should be noted that the time required to find solutions may be prohibitive, especially when considering large data sets. Furthermore, a random process may yield the best solution, but it does not guarantee that it will be acceptable.

## Figures and Tables

**Table 1:** The identity percentage for IIC models is taken into account.

	1	2	3	4	5	6	7
**1**	100	20.8	26.4	36.1	41.7	27.8	33.3
**2**	27.8	100	26.4	20.8	25.0	22.2	25.0
**3**	25.0	23.6	100	27.8	31.9	29.2	26.4
**4**	29.2	30.6	23.6	100	72.2	26.4	29.2
**5**	34.7	26.4	20.8	66.7	100	27.8	33.3
**6**	25.0	20.8	33.3	19.4	20.8	100	23.6
**7**	22.2	19.4	27.8	22.2	25.0	23.6	100

**Table 2:** The identity percentage for CII models is taken into account.

	1	2	3	4	5	6	7
**1**	100	26.4	31.9	23.6	25.0	29.2	27.8
**2**	25.0	100	34.7	29.2	30.6	27.8	25.0
**3**	26.4	30.6	100	37.5	56.9	59.7	22.2
**4**	18.1	29.2	31.9	100	33.3	36.1	18.1
**5**	22.2	26.4	52.8	23.6	100	68.1	19.4
**6**	26.4	25.0	58.3	25.0	73.6	100	26.4
**7**	27.8	26.4	23.6	27.8	25.0	27.8	100

**Table 3:** The statistical characteristics of models obtained with the TF_CII_ target function on validation sets across seven splits are examined.

Split	Characteristics *	All Compounds	Applicability Domain
1	*n*	72	64
	R^2^	0.915	0.911
	MAE	0.259	0.243
2	*n*	72	65
	R^2^	0.923	0.911
	MAE	0.268	0.248
3	*n*	72	59
	R^2^	0.911	0.910
	MAE	0.270	0.249
4	*n*	72	64
	R^2^	0.908	0.915
	MAE	0.208	0.199
5	*n*	72	60
	R^2^	0.919	0.897
	MAE	0.342	0.330
6	*n*	72	60
	R^2^	0.916	0.904
	MAE	0.275	0.245
7	*n*	72	57
	R^2^	0.914	0.925
	MAE	0.296	0.292

* *n* = number of compounds; R^2^ = determination coefficient; MAE = mean absolute error.

**Table 4:** The statistical characteristics of TF_IIC_ models.

Split *	Set	n	R^2^	IIC	CII	Q^2^	Q^2^_F1_	Q^2^_F2_	Q^2^_F3_	<Rm2>	MAE	F	Na
1	A	72	0.5790	0.6808	0.7776	0.5562					0.678	96	
	P	73	0.5958	0.7094	0.7572	0.5738					0.597	105	
	C	72	0.7199	0.8480	0.8261	0.6994	0.7077	0.7046	0.8448	0.5022	0.369	180	
	V	72	0.8271	-	-	-	-	-	-	-	0.33	-	65
2	A	72	0.5745	0.6413	0.7578	0.5548					0.661	95	
	P	73	0.5308	0.6484	0.7194	0.4887					0.726	80	
	C	72	0.6141	0.7836	0.7749	0.5768	0.4927	0.4664	0.8516	0.4879	0.398	111	
	V	72	0.8143	-	-	-	-	-	-	-	0.31	-	66
3	A	72	0.5063	0.5692	0.7069	0.4796					0.739	72	
	P	73	0.3504	0.4667	0.6789	0.2651					0.831	38	
	C	72	0.7323	0.8554	0.8191	0.7193	0.6922	0.6917	0.8951	0.6337	0.313	191	
	V	72	0.8802	-	-	-	-	-	-	-	0.29	-	66
4	A	72	0.4051	0.5694	0.7124	0.3706					0.725	48	
	P	73	0.2548	0.4390	0.7122	0.1438					0.844	24	
	C	72	0.7812	0.8837	0.8533	0.7697	0.7612	0.7526	0.8594	0.6992	0.325	250	
	V	72	0.8927	-	-	-	-	-	-	-	0.26	-	59
5	A	72	0.5168	0.6432	0.7154	0.4911					0.722	75	
	P	73	0.2841	0.4857	0.6717	0.1205					0.933	28	
	C	72	0.7474	0.8645	0.8269	0.7344	0.6771	0.6609	0.8726	0.6521	0.342	207	
	V	72	0.8837	-	-	-	-	-	-	-	0.31	-	58
6	A	72	0.4848	0.6230	0.7366	0.4617					0.716	66	
	P	73	0.4494	0.5761	0.7090	0.4032					0.826	58	
	C	72	0.8585	0.9260	0.8946	0.8510	0.8381	0.8381	0.9140	0.8017	0.282	425	
	V	72	0.8797	-	-	-	-	-	-	-	0.26	-	61
7	A	72	0.4389	0.6267	0.7163	0.4099					0.689	55	
	P	73	0.4609	0.5907	0.7372	0.4262					0.819	61	
	C	72	0.8176	0.9040	0.8772	0.8055	0.8191	0.8068	0.9006	0.7475	0.295	314	
	V	72	0.8566	-	-	-	-	-	-	-	0.30	-	62

* Split = number of corresponding split; A = active training set; P = passive training set; C = calibration set; V = validation set; R^2^ = determination coefficient; IIC = index of ideality of correlation; CII = correlation intensity index; Q^2^ = cross validated correlation coefficient; Q^2^_F1–3_ = the statistical criteria suggested in [32]; <Rm2> = criterion suggested in [33]; MAE = mean absolute error; F = Fischer F-ratio; and Na = the number of active SMILES attributes.

**Table 5:** The statistical characteristics of TF_CII_ models.

Split *	Set	n	R^2^	IIC	CII	Q^2^	Q^2^_F1_	Q^2^_F2_	Q^2^_F3_	<Rm2>	MAE	F	Na
1	A	72	0.6723	0.7336	0.8054	0.6550					0.545	144	
	P	73	0.5558	0.7151	0.7383	0.5348					0.641	89	
	C	72	0.7646	0.6597	0.8463	0.7362	0.6204	0.6204	0.7348	0.6184	0.350	227	
	V	72	0.9152	-	-	-	-	-	-	-	0.26	-	59
2	A	72	0.6499	0.8062	0.8011	0.6335					0.572	130	
	P	73	0.5478	0.7044	0.7534	0.5235					0.730	86	
	C	72	0.9130	0.9382	0.9480	0.9074	0.8277	0.8159	0.8791	0.7380	0.304	735	
	V	72	0.9233	-	-	-	-	-	-	-	0.27	-	64
3	A	72	0.6550	0.7656	0.7953	0.6362					0.520	133	
	P	73	0.5482	0.7272	0.7522	0.5275					0.694	86	
	C	72	0.8541	0.6901	0.8961	0.8434	0.8047	0.7720	0.8181	0.7480	0.347	410	
	V	72	0.9119	-	-	-	-	-	-	-	0.27	-	65
4	A	72	0.6678	0.7730	0.8064	0.6512					0.596	141	
	P	73	0.5650	0.7032	0.7677	0.5449					0.718	92	
	C	72	0.8757	0.9283	0.9229	0.8670	0.8791	0.8667	0.9377	0.8254	0.238	493	
	V	72	0.9077	-	-	-	-	-	-	-	0.21	-	60
5	A	72	0.6754	0.7774	0.8061	0.6579					0.585	146	
	P	73	0.6066	0.6214	0.7964	0.5873					0.678	109	
	C	72	0.8886	0.6726	0.9406	0.8808	0.8123	0.7493	0.9070	0.7003	0.299	558	
	V	72	0.9188	-	-	-	-	-	-	-	0.34	-	65
6	A	72	0.6172	0.7029	0.8085	0.5982					0.631	113	
	P	73	0.5309	0.5447	0.7634	0.5078					0.689	80	
	C	72	0.8621	0.4806	0.9215	0.8484	0.7650	0.7636	0.8060	0.7515	0.329	438	
	V	72	0.9163	-	-	-	-	-	-	-	0.28	-	61
7	A	72	0.7256	0.7621	0.8217	0.7126					0.566	185	
	P	73	0.4994	0.6450	0.7616	0.4772					0.709	71	
	C	72	0.8548	0.7689	0.8973	0.8461	0.7506	0.7503	0.8469	0.6601	0.321	412	
	V	72	0.9138	-	-	-	-	-	-	-	0.30	-	59

* Split = number of corresponding split; A = active training set; P = passive training set; C = calibration set; V = validation set; R^2^ = determination coefficient; IIC = index of ideality of correlation; CII = correlation intensity index; Q^2^ = cross validated correlation coefficient; Q^2^_F1–3_ = the statistical criteria suggested in [32]; <Rm2> = criterion suggested in [33]; MAE = mean absolute error; F = Fischer F-ratio; and Na = the number of active SMILES attributes.

**Table 6:** Lists of promoters of increase or decrease in sweetness (split-1, TF_CII_ optimization).

	Structural Attributes	Run 1	Run 2	Run 3	NA	NP	NC	Statistical Defect
Increase	NNC-C...321.	0.4769	0.4176	0.5039	70	71	70	0.0000
	NNC-C...220.	0.4550	0.0824	0.0032	62	60	62	0.0004
	NNC-O...220.	1.3223	0.4659	1.0529	60	65	59	0.0008
	=...........	0.1880	0.5179	0.4768	55	55	60	0.0009
	=...(.......	0.6840	1.4828	1.3081	51	53	57	0.0010
	NNC-C...211.	1.0089	1.2289	1.2140	50	52	55	0.0009
	NNC-C...110.	0.8197	0.0149	0.1918	49	40	45	0.0020
	N...........	1.0684	2.0239	1.1876	42	43	42	0.0001
	NNC-N...220.	3.5593	4.1084	4.4190	39	39	36	0.0007
	C...1.......	1.2053	1.6808	0.6510	38	38	38	0.0001
	NNC-C...101.	0.6648	0.7533	0.6498	18	20	16	0.0019
	[C@@].......	2.0478	1.0933	1.6548	14	19	13	0.0035
	[C@]........	1.5196	2.5803	2.1728	11	9	10	0.0020
	O...2.......	1.1305	1.3308	1.3652	10	8	7	0.0033
	NNC-C...440.	0.9277	0.1667	0.5834	10	8	12	0.0038
Decrease	O...........	−0.4200	−0.4548	−0.6869	72	73	72	0.0000
	O...(.......	−0.2848	−0.3945	−0.7007	71	72	71	0.0000
	NNC-O...110.	−0.3567	−1.2333	−1.0353	71	72	71	0.0000
	C...C.......	−0.2592	−0.1303	−1.1669	58	54	58	0.0008
	N...(.......	−1.5521	−0.6209	−1.0185	42	43	40	0.0005
	NNC-C...330.	−0.0827	−0.1259	−0.5471	41	35	40	0.0016
	N...C.......	−4.3522	−4.7335	−5.3242	40	40	36	0.0010
	O...1.......	−0.2392	−0.6323	−0.6564	24	27	25	0.0010
	3...........	−1.8613	−2.2300	−2.0569	10	9	10	0.0011

**Table 7:** Comparison of sweetness models.

Number in Training Set	R^2^ for Training Set	Number in Validation Set	R^2^ for Validation Set	Reference
≈318	0.89	≈137	0.83	[14]
36	0.96	7	0.57	[15]
236	0.74	79	0.87	[29]
217	0.67	72	0.91	In this study (split-1, TF_CII_)

## Data Availability

The original contributions presented in this study are included in the article and Supplementary Materials. Further inquiries can be directed to the corresponding author.

## Supplementary Materials

The following supporting information can be downloaded at: https://www.mdpi.com/article/10.3390/foods15091481/s1. Table S1 contains technical details on the model for split 1 with TF_IIC_-optimization; Table S2 contains technical details on the model for split 1 with TF_CII_-optimization.

## Abbreviations

The following abbreviations are used in this manuscript:

QSPR	Quantitative structure–property relationships
QSAR	Quantitative structure–activity relationships
SMILES	Simplified molecular input-line entry system
CW	Correlation weight
DCW	Optimal descriptor of correlation weights
NNC	Nearest neighbor code
IIC	Index of ideality of correlation
CII	Correlation intensity index
